# The evolutionary rate variation among genes of HOG-signaling pathway in yeast genomes

**DOI:** 10.1186/1745-6150-5-46

**Published:** 2010-07-10

**Authors:** Xuechang Wu, Xiaoqin Chi, Pinmei Wang, Daoqiong Zheng, Rui Ding, Yudong Li

**Affiliations:** 1 Institute of Microbiology, College of Life Sciences, Zhejiang University, Hangzhou 310058, PR China

## Abstract

**Background:**

Responses to extracellular stress are required for microbes to survive in changing environments. Although the stress response mechanisms have been characterized extensively, the evolution of stress response pathway remains poorly understood. Here, we studied the evolution of High Osmolarity Glycerol (HOG) pathway, one of the important osmotic stress response pathways, across 10 yeast species and underpinned the evolutionary forces acting on the pathway evolution.

**Results:**

Although the HOG pathway is well conserved across the surveyed yeast species, the evolutionary rate of the genes in this pathway varied substantially among or within different lineages. The fast divergence of *MSB2 *gene indicates that this gene is subjected to positive selection. Moreover, transcription factors in HOG pathway tend to evolve more rapidly, but the genes in conserved MAPK cascade underwent stronger functional selection. Remarkably, the *d*_N_/*d*_S _values are negatively correlated with pathway position along HOG pathway from Sln1 (Sho1) to Hog1 for transmitting external signal into nuclear. The increased gradient of selective constraints from upstream to downstream genes suggested that the downstream genes are more pleiotropic, being required for a wider range of pathways. In addition, protein length, codon usage, gene expression, and protein interaction appear to be important factors to determine the evolution of genes in HOG pathway.

**Conclusions:**

Taken together, our results suggest that functional constraints play a large role in the evolutionary rate variation in HOG pathway, but the genetic variation was influenced by quite complicated factors, such as pathway position, protein length and so on. These findings provide some insights into how HOG pathway genes evolved rapidly for responding to environmental osmotic stress changes.

**Reviewers:**

This article was reviewed by Han Liang (nominated by Laura Landweber), Georgy Bazykin (nominated by Mikhail Gelfand) and Zhenguo Lin (nominated by John Logsdon).

## Background

Sensing and responding to their changing environmental conditions are crucial challenges for microbes to survive in highly divergent niches they occupy [1,2]. The effective responses to different stresses are mediated by stress signaling pathways, which are generally composed of three functional modules: reception module, transduction module and response module [1,3]. The HOG (High Osmolarity Glycerol)-MARK (Mitogen Activated Protein Kinase) pathway is required for adapting to osmotic stress in yeast and other eukaryotes [4,5].

**Figure 1:**
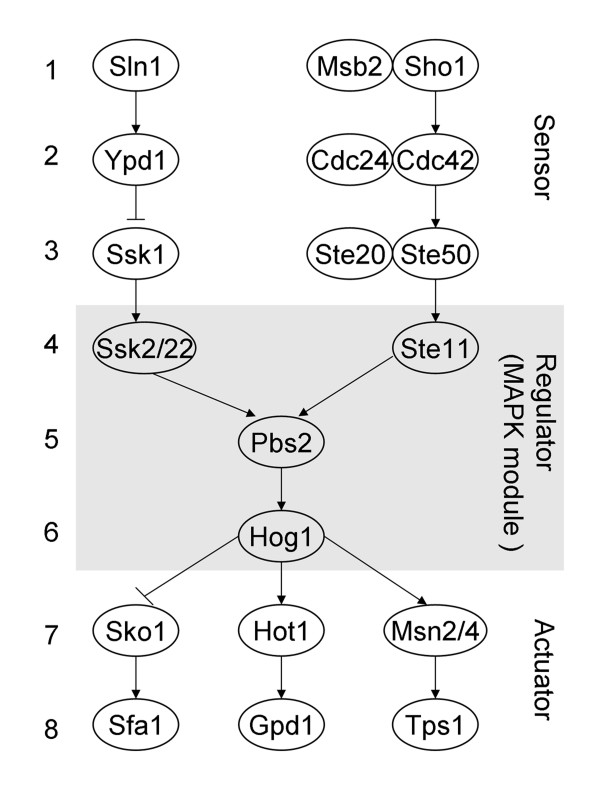
**An illustration of the HOG-MAPK pathway genes in *S. cerevisiae***. Arrows indicate the direction of signal transduction. The numbers on the left side represent the position of the HOG pathway genes.

The HOG pathway is greatly characterized in budding yeast *Saccharomyces cerevisiae *[6,7]. The Hog1 (stress activated MAP kinase) is the central component of the pathway, and is regulated by Pbs2 (MAPK kinase). In turn, the Pbs2 activity is controlled by two independent osmosensing branches involving two transmembrane proteins Sho1 and Sln1, respectively (Figure 1). The two upstream branches of the HOG pathway are functionally redundant in some respects, but they use different components and seem to have slightly different sensitivities to the concentration of external solutes [8]. In response to hyperosmotic stress, Pbs2 becomes activated, leading to the phosphorylation and nuclear accumulation of Hog1, and the subsequent activation of osmo-protective mechanisms, such as the accumulation of the glycerol [9].

Fungi, one of the three main eukaryotic kingdoms, are excellent models to study environmental stress responses pathways, because they are simple and evolutionarily conserved [1,10]. Fungi display significant variations in their stress resistance to osmotic stress. For example, the human pathogen, *Candida albicans*, tends to be resistant to osmotic stress, while the plant pathogen, *Ashbya gossypii*, is relatively sensitive to osmotic stress [10]. This phenomenon reflects their adaptive evolution in specific niches where they have been threatened by different stresses. Recent researches have proved that fungi stress signaling pathways have evolved rapidly in a niche-specific fashion, and the evolution of signaling pathway is independent of fungi's phylogenetic relationship [1,10]. The availability of many genome sequences from related yeast species allows us to conduct the evolutionary analysis of the entire pathway in these species.

The evolution of individual genes or gene families has been studied extensively, and many factors have been found to determine the evolution of genes, such as protein length, codon usage, gene expression, and protein interaction [11-13]. Recently, a few researches have found that the position of the genes in metabolic pathway affected their evolutionary rate, and the upstream elements tended to evolve faster than the downstream elements [14]. In contrast, several studies also showed that central components of signaling pathways were generally well conserved, whereas upstream sensors and downstream transcriptional regulators had diverged to a greater extent [1,10]. However, the mechanisms underlying the pathway gene evolution remain largely unknown. Therefore, more pathways with different topologies are needed to disentangle the underlying evolutionary forces that affect the molecular evolution pattern of the elements in pathways [15]. The HOG pathway, which has specific topology with two input branches, provides an excellent system for exploring the mechanisms of pathway evolution.

In this study, we studied the molecular evolution of the HOG pathway genes and the relationship between evolutionary rates with pathway position. Our results showed that the functional constraints play a large role in the overall rate variation in HOG pathway, and the position of gene in pathway was negatively correlated with evolutionary rate. In addition, the pathway evolution may also be influenced by protein length, codon usage and gene expression.

## Results and Discussion

### The distribution of HOG pathway genes in yeast genomes

According to the well-characterized HOG-signaling pathway [1,7], we identified 19 HOG-MAPK pathway genes from *S. cerevisiae *to be analyzed in our study (Figure 1). The initial set of *S. cerevisiae *HOG-signaling pathway genes were used to identify candidate orthologs in the genomes of remaining yeast species, and a total of 203 putative orthologs in 10 yeast genomes were identified (Table 1). Generally, the orthologs identified in this study were consistent with that of Krantz et al. [7], despite that we used the method based on gene synteny.

**Table 1:** Copy number of the HOG signaling pathway genes in 10 fungi genomes

Gene	*Scer*	*Spar*	*Smik*	*Sbay*	*Scas*	*Agos*	*Kwal*	*Klac*	*Cgla*	*Calb*
*SLN1*	1	1	0/1^a^	0	1	1	1	1	1	1
*YPD1*	1	1	1	1	2	1	1	1	1	1
*SSK1*	1	1	1	1	1	1	1	1	1	1
*SSK2/22*	2	1	1^b^	2	2^b^	1	1	1	1	1
*PBS2*	1	0/1	0/1	1	1^b^	1	1	1	1	1
*HOG1*	1	0/1	0/1	1	1	1	1	1	1	1
*HOT1*	1	0/1	1	1	1	1	1	1	1	2
*GPD1*	2	1	0/1	2	2	1	1	1	2	1
*SHO1*	1	1	1	1	1	1	1	1	1	1
*MSB2*	1	1	1	1	1	1	1	1	1	1
*CDC42*	1	0/1	0/1	1	1	1	1	1	1	1
*CDC24*	1	1	1	1	1	1	1	1	1	1
*STE20*	1	1	1	1	2	1	1	1	2	1
*STE50*	1	1	1	1	1	1	1	1	1	1
*STE11*	1	1	1	0	1	1	1	1	1	1
*SKO1*	1	1	1	1	1	1	1^b^	1	1	1
*MSN2/4*	2	1	1	1	2	1	1	1	2	1
*TPS1*	1	1	1	1	2	1	1	1	1	1
*SFA1*	1	1	1	1	1	1	1	1	1	0/1

^a ^"0/1" represents the orthologs were identified by BLAST search.

^b ^DNA sequence included stop codons.

**Figure 2:**
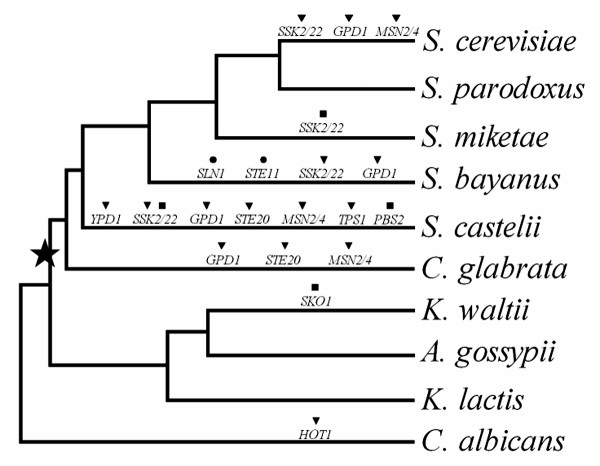
**The phylogenetic tree showing the evolutionary relationships of the 10 yeast species studied**. The black star shows the timing of the whole genome duplication (WGD). Gene duplication (black triangle), loss (black circle), and pseudogenization (black square) events detected in the HOG pathway across the yeast species were shown above each branch.

The HOG-MAPK pathway is well conserved across the available genomes of 10 yeast species. Most genes have orthologs in all the yeast species studied and were identified as unique singletons in most cases. Together, 8 HOG pathway genes had a 1:1 orthology relationship, while the remaining 11 genes underwent a number of duplication or loss events (15 duplications, 2 loss, and 4 pseudogenization events; Figure 2). The loss or pseudogenization of orthologs may be due to the uncompleted, low quality genome sequencing of the species, because partial sequences of the lost orthologs were found in the species' contig sequence by BLAST searching. The genes whose ortholog were lost (*SLN1 *and *STE11*) or included stop codons (*SSK2, PBS2 *and *SKO1*) in a given species were not used in further analysis.

With the 8 conserved 1: 1 orthologs, we build the phylogenetic tree of yeast species by Bayesian method (Figure 2), and the topology of the phylogenetic tree is consistent with previous study [16]. As *Saccharomyces *species are derived from an ancestral yeast that underwent a whole genome duplication (WGD) [17], the gene duplication events we observed in HOG pathway mostly occurred in post-WGD species (Figure 2).

### The variation of *d*_N_/*d*_S _values among genes of the HOG pathway

**Figure 3:**
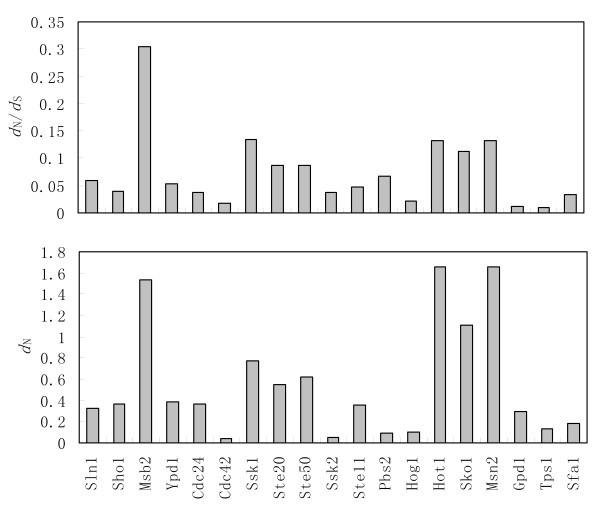
**Maximum likelihood estimates of the rates of amino acid replacements (*d*_N_) and synonymous changes (*d*_S_) across the phylogenetic tree**. The genes are listed in order of their relative position within the pathways.

The natural selection acting on genes is inferred by the ratio of nonsynonymous substitutions (*d*_N_)/synonymous substitutions (*d*_S_). By using M0 model, a single *d*_N_/*d*_S _value for all lineages was estimated for each gene in the HOG pathway. The result show that divergence across species is not uniformly distributed along the pathway, but varies greatly among genes (Figure 3). Within sensor module genes, there is a 17-fold difference in the level of divergence between the most conserved (*CDC42*) and the most divergent (*MSB2*) gene. Moreover, the pattern of divergence exhibited by *d*_N _values precisely matches the pattern of *d*_N_/*d*_S _value (Figure 3).

The estimates of the *d*_N_/*d*_S _ratio, using the free-ratio model, also show that the *d*_N_/*d*_S _values of the HOG pathway genes vary substantially in each lineage, ranging from 0.0001 (*CDC42 *of Smik) to 1.19 (*MSN2 *of Calb). However, the mean *d*_N_/*d*_S _value equals 0.196, suggesting strong functional constraints acting at most of the HOG pathway genes. The very high degree of divergence observed at the *MSB2 *gene indicates that this gene is subjected to positive selection. By using the maximum likelihood method to detect positive selection, we identified gene *MSB2 *may experience positive selection (LRT = 35.72, p < 0.001). As this gene is located at the start point of the HOG pathway and involved in sensing the environmental changing, it tends to undergo positive selection [18].

### Regulatory genes exhibit higher levels of divergence, but MAPK cascade genes are highly conserved

Recently, transcription factors have been reported to show evidence of rapid evolution [19,20]. We also observed that transcription factors Hot1, Msn2 and Sko1, at the position 7 of the pathway, evolve substantially faster than their surrounding genes in HOG pathway (Figure 3). Only one structural gene *MSB2*, which has been identified as positively selected gene, show comparable levels of amino acid replacement changes. On average, the transcription factors are more divergent than the structural genes (Wilcoxon rank sum test, *P *= 0.014). The higher levels of divergence can be either the result of relaxed selection and accumulation of neutral or slightly deleterious substitutions or the result of positive selection leading to the fixation of beneficial mutations [13,19]. However, we do not find any evidence of positive selection on these transcription factors by using the maximum likelihood methods (see Methods).

**Figure 4:**
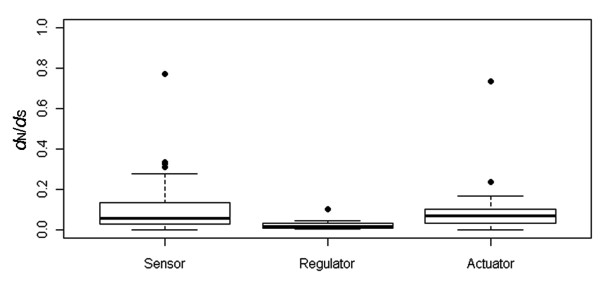
**Box plot of the *d*_N_/*d*_S _values of the HOG pathway modules in yeast species**. The difference between regulator module (MAPK cascade) and other modules was significantly different (Wilcoxon rank sum test, *P *= 0.001154 for sensor module, *P *= 0.000539 for actuator module). Dots represent the *d*_N_/*d*_S _values, and black line respresent the mean value.

According to an engineering view of dynamical control, gene function in HOG-signaling pathway can be classified into three functional roles: "sensor", "regulator", "actuator" (Figure 1) [3,21]. The *d*_N_/*d*_S _values of the MAPK cascade genes (regulator module) was significantly smaller than upstream sensor module (Wilcoxon rank test, *P *= 0.00115) and downstream module (Wilcoxon rank test, *P *= 0.000539) (Figure 4). This is consistent with the view that the MAPK cascade is highly conserved and under stronger functional constraints, because it's involved in many other processes ranging from mating to invasive growth [22].

### The selective constraints was correlated with pathway position of those genes in signal transduction process

**Figure 5:**
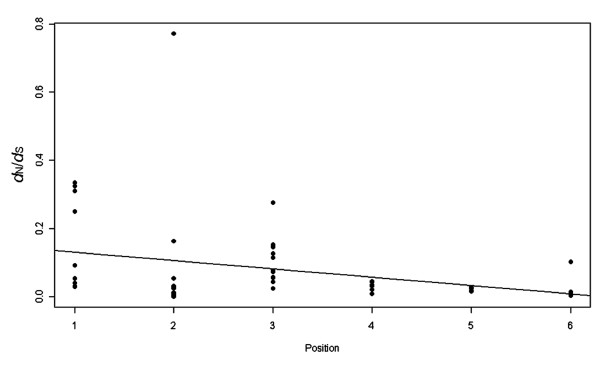
**Correlation between the *d*_N_/*d*_S _value and the position during the HOG signal transduction process from Sln1 (Sho1) to Hog1**. The position of the HOG pathway genes was as shown in Figure 1. The downstream elements have higher levels of selective constraint than the upstream elements (Spearman's rank correlation coefficient: ρ = -0.313, *P *= 0.0189).

Several earlier studies have shown that the architecture of pathway, in particular, pathway position, shapes the evolution of its genes [14,23,24]. However, how the topology of the pathway affects the pathway evolution remains elusive. During the HOG signal transduction process, the external osmotic stress was sensed by Sln1 and Sho1, and the signal was propagated via a MAPK cascade that finally caused nuclear import of Hog1 [22]. As shown in Figure 5, variation of *d*_N_/*d*_S _was correlated significantly with pathway position in the HOG-signaling pathway (Spearman's rank correlation coefficient: ρ = -0.313, *P *= 0.0189). However, the relationship does not hold if including these genes in position 7 and 8 of HOG pathway. For signal transduction pathway receive signaling inputs from a number of pathways (i.e., at least two branches Sln1 and Sho1 of HOG pathway) and share single response output (MAPK module), the downstream genes are expected to be more pleiotropic, and hence be subjected to greater selective constraint than the upstream genes. Therefore, the pathway position correlated with functional constraint levels can be explained by the information flux theory that the elements located at branch point of pathway exert a greater flux control and subjected to stronger selective constraints [14,23].

Even the pooled *d*_N_/*d*_S _data was separated into each of the pathway branches Sln1 and Sho1, they also displayed the same trends (Spearman's rank correlation coefficient for Sln1: ρ = -0.427, *P *= 0.0261; for Sho1: ρ = -0.412, *P *= 0.00833). However, the position of genes in Sho1 branch was not clearly determined, especially position 2 (*CDC24 *and *CDC40*) and position 3 (*STE20 *and *STE50*) [5,25]. Although we changed the positions of these genes, the evolutionary pattern was not affected by their related locations. What's more, if these data were separated into each lineage of the surveyed yeast taxa, the pattern of *d*_N_/*d*_S _variation correlated significantly with pathway position still holds in *S. parodoxus*, *S. miketae *and *A. gossypii*. The lack of coordinated evolution in each lineage had been observed in anthocyanin and terpenoid biosynthetic pathway genes among plant lineages [23,24].

### The other factors affecting the evolution of HOG pathway genes

The selective constraints on the pathway genes are also affected by other evolutionary factors. We studied the association between molecular evolution of the genes in HOG-MAPK pathway with codon usage, gene expression, protein length, and protein interaction in *S. cerevisiae *(Table 2). Selective constraint on genes was measured as ω (*d*_N_/*d*_S_) and codon bias was measured as the frequency of optimal codon (*F*op). Using simple Spearman rank correlation analyses, the ω (*d*_N_/*d*_S_) shows significant correlated with codon bias (ρ = -0.578, *P *= 0.00959) and protein length (ρ = 0.47, *P *= 0.0426), whereas not significantly associated with other factors. Generally, natural selection is expected to affect codon usage, and higher codon bias indicates stronger constraints acting on a gene [13]. An earlier study have also documented that the protein length play an important role in shaping the evolution of proteins in *P. tremula *[12].

**Table 2:** Summary statistics used in the multivariate analysis

Gene	Position	**Protein length**^a^	*d*_N_^b^	ω^b^	PPI^c^	*F*op^c^	Eavg^c^
*SLN1*	1	1220	0.0156	0.0534	23	0.435	1784.290
*YPD1*	2	167	0.000014	0.0001	31	0.534	1017.443
*SSK1*	3	712	0.0208	0.0767	31	0.414	2416.731
*SSK2*	4	1579	0.00758	0.0316	29	0.445	2156.276
*PBS2*	5	668	0.00731	0.0276	45	0.456	1829.891
*HOG1*	6	435	0.00122	0.0057	82	0.487	3725.510
*HOT1*	7	719	0.0364	0.109	16	0.407	1135.488
*GPD1*	8	391	0.00115	0.0072	5	0.744	7653.448
*SHO1*	1	367	0.00702	0.0298	90	0.463	1998.004
*MSB2*	1	1306	0.0677	0.2496	20	0.457	5044.198
*CDC24*	2	854	0.0028	0.0093	78	0.442	2105.747
*CDC42*	2	191	0.0049	0.011	113	0.495	6086.679
*STE20*	3	939	0.0161	0.0748	141	0.463	1619.444
*STE50*	3	346	0.0155	0.1138	61	0.464	1441.888
*STE11*	4	717	0.0118	0.0425	105	0.443	1512.138
*SKO1*	7	647	0.0253	0.0676	19	0.408	2101.820
*MSN4*	7	630	0.0108	0.0467	10	0.462	1855.669
*SFA1*	8	386	0.00923	0.0303	11	0.553	3738.413
*TPS1*	8	495	0.000025	0.0001	60	0.549	7123.245

^a ^Number of amino acids in the *S. cerevisiae *protein.

^b ^The *d*_N _and ω values in the *S. cerevisiae *lineages.

^c ^PPI: protein and protein interaction; *F*op: Frequency of optimal codons; Eavg: Average gene expression levels.

**Figure 6:**
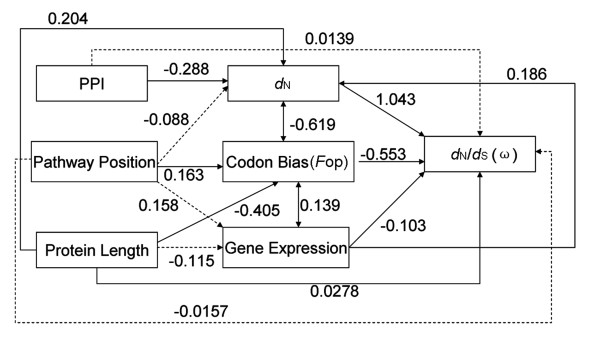
**Graphical representation of the path analysis used to analyze the relationships among pathway positions, nonsynonymous substitution (*d*_N_), *d*_N_/*d*_S _ratio, gene expression level, codon bias (measured by *F*op), protein length and protein interaction**. Pathway position, protein length and connectivity were treated as exogenous variables, while the rest were treated as endogenous variables. The numbers on the arrows represent the standardized path coefficients (β). Solid and dotted lines represent significant and non-significant relationships, respectively.

However, these factors are inter-correlated, and some of the observed correlations might actually result from indirect effects rather than from direct effects. Path analysis was used to better characterize the direct and indirect effects of these factors. As shown in Figure 6, *d*_N_/*d*_S _show a significant correlation with codon bias, protein length and gene expression. The position do not correlate with *d*_N_/*d*_S _directly, thus their relationship might actually result from indirect effects, connected by codon usage_. _Nevertheless, these factors affecting protein evolution in HOG pathway are complex, with interplay of a large number of factors.

## Conclusions

The availability of complete genome sequences from many fungal species presents a good opportunity to examine the evolution of pathway genes. In this study, we investigated the genetic variation of HOG-signaling pathway genes across 10 yeast species and underpinned the evolutionary forces acting on the pathway evolution. Our results showed that nearly all the pathway genes existed in the surveyed species and subjected to relatively high selective constraints, suggesting that the HOG pathway is functional across all these species. However, the high levels of evolutionary rate variation of HOG pathway genes among and within different lineages are consistent with the hypothesis that stress signaling pathways have evolved rapidly in a niche-specific fashion [1,10].

As the signal pathway could be grouped into three functional modules (sensor, regulator, actuator) [1,6], the most dramatic variation in HOG pathway was concentrated to the evolution of sensor genes (Figure 3). Since these genes are involved in sensing environmental changing, their high genetic variation was possibly due to adaptive evolution. For instance, *MSB2 *was identified as positively selected genes. In contrast, the MAPK cascade was the least variation module in the HOG pathway, which is consistent with the view that it is well conserved in eukaryotes, and involves in multiple stress tolerance processes (salt, H_2_O_2_, low and high temperature) [4,22].

Furthermore, the position of the genes in HOG pathway significantly correlated with their *d*_N_/*d*_S _value variation. Some recent studies also have demonstrated that selective constraint levels are correlated with the position of the elements in metabolic pathways or signal pathways [14,24]. Signal transduction pathways receive signals from many other pathways and transmit them into single output (i.e., Hog1 in HOG pathway) to activate transcription reprogramming. Therefore, there are stronger selective constraints acting on the downstream genes than the upstream ones. The evolution pattern of HOG pathway supported that natural selection primarily targets enzymes that have the greatest control on fluxes [14,24].

Notably, the transcription factors in HOG pathway tend to evolve faster, which is consistent with a large number of recent studies [19,20]. To respond sensitively to the change of environment, stress response pathways are composed of intricate gene regulation networks [20]. Generally, transcription factors tend to be the hub node in regulation network and under stronger functional constraints. However, the fast evolution of transcription factors in HOG pathway may be the result of their central roles in controlling regulation network to adapt with the changing of environmental pressures. Moreover, the evolutionary rate variation of the HOG pathway was correlated with codon bias, protein length and gene expression, suggesting that the genetic variation may be influenced by quite complicated factors. Nevertheless, further work will be needed to assess the generality of the pathway evolution pattern we observed in yeast species.

## Methods

### Identification of HOG pathway genes in yeast genomes

The HOG pathway genes of *S. cerevisiae *and their interaction (Figure 1) were generated according to Bahn *et al*. (2007) and Krantz *et al*. (2006) [1,7,26]. The full list of HOG pathway genes included in this study is presented in Table 1. There are 10 yeast species were used in our study: 6 post-whole genome duplication (WGD) species (*S. cerevisiae *[Scer], *S. parodoxus *[Spar], *S. miketae *[Smik], *S. bayanus *[Sbay],*S. castelii *[Scas], and *Candida glabrata *[Cgla]), and 4 pre-WGD species (*Kluyveromyces waltii *[Kwal], *K. lactis *[Klac], *Ashbya gossypii *[Agos], and *C. albicans *[Calb]). The protein coding sequences (CDS) of the HOG pathway genes in the *S. cerevisiae *genome were retrieved from *Saccharomyces *Genome Database (SGD) [27]. Orthologous sequences of these genes in *Candida albicans *were obtained from CGD http://www.candidagenome.org[28], and in other species were obtained from Yeast Gene Order Browser website http://wolfe.gen.tcd.ie/ygob/[29] or the yeast comparative genomics website http://www.broadinstitute.org/annotation/fungi/comp_yeasts[30]. These orthologs were curated based on gene positional synteny and sequence similarity. For those genes without ortholog annotation, their orthologs were identified based on the Reciprocal BLAST best hits between *S. cerevisiae *and other species, with an *E*-value cutoff of 10^-6^, an identity = 40% and at least 75% alignable region [31]. All sequence alignments are available on request.

### Sequence alignment and phylogenetic reconstruction

We generated a multiple sequence alignment (MSA) of the protein sequences of each orthologs using the software Clustalw 2.01 [32]. The resulting alignments were manually improved using the software bioedit 7.0.9 [33]. The aligned amino acid sequences were reversely converted into nucleotide sequences by in-house Perl script for further analysis. In the cases in which there was more than one gene copy in a given species, we used the gene with the same synteny location. The orthologs with stop codons were not used in further analysis (see Table 1).

The genes with 1:1 orthologs in all the species were concatenated for building consensus phylogenetic tree [34]. We conducted a bayesian phylogenetic reconstruction using the software MrBayes 3.1.2 [35], applying the nucleotide substitution model that best fits the data according to the Akaike information criterion. The FindModel program http://hcv.lanl.gov/content/sequence/findmodel/findmodel.html was used for model selection, and the best-fitting model was the GTR+Γ model. All analyses were conducted allowing for a proportion of sites to be invariable (I). The phylogenetic tree was used for further analysis.

### Estimation of Evolutionary rate and detection of positive selection

We evaluated the impact of natural selection by estimating nonsynonymous (*d*_N_) and synonymous substitution (*d*_S_), and their ratio (ω = *d*_N_/*d*_S_) using the program codeml of the PAML 4.1b package [36]. The M0 model (which assumes a single ω value for all lineages) and M1 model (to give separate *d*_N_/*d*_S _values for each lineage), were used for evolutionary analyses, with cleandata = 1 (remove sites with ambiguity data) and get SE = 1 (obtain standard errors of ω estimates) [18]. Saturation effects were avoided by discarding pairwise gene comparisons for which *d*_S _> 2 and *d*_S _< 0.005 [24]. We also restricted this analysis to the five *Saccharomyces *group species to avoid saturation at synonymous sites, which could bias the *d*_N_/*d*_S _estimates because the sequence alignments of these 5 species were more reliable than that of all species.

To determine whether there are codons evolving under positive selection, we compared the M7 and M8 models using the likelihood ratio test, which had been shown to be more powerful than other branch-site models [18,37]. The Bayes Empirical approach was used to identify the codons evolving under positive selection (posterior probability = 95%). We conducted all likelihood estimations using three different ω starting values (0.01, 0.3 and 3) to overcome the problem of multiple local optima. All these analyses were conducted using the F3x4 codon frequency model [38]. We corrected for multiple testing by using false discovery rate (FDR) of 0.05 for these analyses.

### Statistic analysis

All statistical analyses were performed by using statistical package R http://www.r-project.org. The statistical significance for the difference was determined by Wilcoxon rank sum test and the Spearman rank correlation was used for correlation analysis. The influence of parameters, such as expression level, codon bias, protein length, and protein interaction (PPI), on evolutionary rate was analyzed in this study [39]. Protein length, expression level and codon usage were obtained from the *Saccharomyces *Genome Database and references [27,39]. The number of PPIs was obtained from GRID (General Repository for Interaction Datasets). First, we evaluated whether these parameters are correlated using Spearman's rank correlation coefficient (ρ). Later, we analyzed the data using path analysis, an extension of multiple regression analysis that allows decomposing the regression coefficients into their direct and indirect components by considering an underlying user-defined causal model [14]. Before performing path analysis, these data were log-transformed to improve normality. The path analysis was performed using the sem package in R.

## Abbreviations

HOG: High Osmolarity Glycerol; WGD: Whole Genome Duplication; LRT: Likelihood ratio test; *F*op: Frequency of optimal codons; PPI: Protein and protein interaction; MSA: Multiple sequence alignment.

## Competing interests

The authors declare that they have no competing interests.

## Authors' contributions

XCW and YDL designed the research and wrote the manuscript together; XQC, PMW, DQZ and RD participated in the analyses and helped to draft the manuscript. All authors have read and approved the final manuscript.

## Reviewer' comments

### Reviewer's report 1

#### Han Liang, Department of Bioinformatics & Comp Biology, University of Texas M. D. Anderson Cancer Center (nominated by Laura Landweber)

Understanding the evolutionary patterns in the context of biological pathways is a topic of great importance. Wu and colleagues performed a comprehensive analysis on a well-characterized pathway in yeasts, HOG-signaling pathway. They found that the evolutionary rate of different components shows great variation, and the variation is influenced by many factors. This is an interesting study, providing useful insights into the evolution of a key pathway.

My comments are as follows:

1. As for the distribution of HOG pathway genes in the yeast phylogeny, does the gene duplicability (i.e., gene copy gain or loss) correlate with the pathway position?

Author's response: *There are no correlation was observed between pathway position and gene duplicability. We observed the gene duplication events in HOG pathway mostly occurred in post-WGD species*.

2. The authors detected positive selection on *MSB2 *gene. Is it possible to pinpoint the amino acids under adaptation and infer the functional effect of the amino acid changes?

Author's response: *Thanks for the valuable suggestion. Besides the fast evolution of MSB2 gene, we also find that there is large insert/deletion in some strains of Saccharomyces cerevisiae. We will investigate the functional effect of these sequence changes in future work*.

3. The authors reported that the MAPK genes are under stronger functional constraints and that the evolutionary rate is negatively correlated with the positions in the pathway (positions 1-6). These two points are actually redundant because the MAPK (regulator module) is in the downstream of sensor module. So within a module, does the evolutionary rate depend on the position?

Author's response: *According to an engineering view of dynamical control, gene function in a signaling pathway can be classified into three functional roles: "sensor", "regulator", "actuator". In each module, the downstream elements have higher levels of selective constraint than the upstream elements. For regulator module, ρ = -0.89, P = 0.006 (Spearman's rank correlation). The correlation of other two modules is more clearly *(Figure 3).

4. In the last section, the authors examined the correlations of *d*_N_/*d*_S _with a series of factors. Since these factors are often interrelated, it would be better to use principal component analysis or partial correlation analysis to estimate the relative contributions of each factor?

Author's response: *We agree with the reviewer that the suggested statistical analysis should be better to estimate the relative contributions of each factor. In this study, we used simple Spearman rank correlation analysis to estimate the correlation, and the path analysis to estimate the indirect effects of these factors. Therefore, we tried to findout which factors affect the evolution, but not considering which factor play main role in the evolution*.

Minor points:

"Most genes have orthologs in all the yeast species and were identified as unique singletons in most cases." How can 8 out of 19 genes become "most"?

Author's response: *There are 10 yeast species surveyed in this study. The total number of genes is 190. As shown in *Table 1, *the singleton gene number is 169 (190 - (15 + 2 + 4)). So we say that most genes were singleton*.

I declare that I have no competing interests.

### Reviewer's report 2

#### Georgy Bazykin, Department of Bioinformatics, Russian Academy of Science (nominated by Mikhail Gelfand)

The manuscript by Wu et al. is an addition to the existing literature on correlations between the pathway position of the gene and its evolutionary rate. The authors show that the genes located downstream in HOG pathway are under stronger negative selection.

My main concern is that there seems to be a number of flaws in the statistical analyses supporting the authors' main conclusions.

1. Multiple testing. The authors test all 19 genes of the HOG pathway with the free-ratio model of codeml (apparently for a total of ~19*7 comparisons), and identify a single gene at a single branch (*MSN2 *of Calb) with *d*_N_/*d*_S _> 1. They then test *MSB2 *gene for positive selection using LRT, and detect positive selection. However, due to the sheer number of multiple tests involved, detecting a single gene with *d*_N_/*d*_S _> 1 could occur due to chance effects. There is no built-in correction for multiple testing in codeml (Yang 1998 Mol Biol Evol 15(5):568-573), and the results of multiple comparisons should be interpreted with more caution. In Methods, the authors write that they used the FDR rate of 0.05, but this apparently only applies to comparisons of codons using M7 and M8 models (which apparently is never implemented, see below).

Author's response: We agree with the reviewer, and as this comment says, we first test all 19 genes of the pathway with the free-ratio model, then identify positive selection using likelihood ratio test (LRT) test to compare M7 and M8 model. However, the gene with d_N_/d_S_>1 is not used as evidence to detect positive selection, which is applied by the comparisons of codons using M7 and M8 models in this study. We have described this analysis in Methods (Estimation of evolutionary rate and detection of positive selection). Furthermore, considering 1 out of 19 tests to be positive, and FDR cutoff = 0.05, the P value should be P < 0.0026 (1 * 0.05/19). As the MSB2 with P < 0.001, so it's selected as positive selection gene.

2. Arbitrariness in selection of data. In analysis of the correlation between the pathway position and *d*_N_/*d*_S_, the authors exclude genes in position 7 and 8 of HOG pathway. Why these genes should be excepted is not explained, and the authors include them in the rest of the tests.

Author's response: *The exception of position 7 & 8 could be explained by two reasons: first, it made sense that the external signal is transmitted into nuclear from Sln1/Sho1 to Hog1, which changed gene transcription. The HOG signal transduction process is restricted in genes from SLN1 to HOG1, but transcription factors are involved in many other biological processes. Second, the increased divergence of transcription factors in postion 7 had been observed. If the position 7 and 8 was included, the correlation didn't hold again*.

I declare that I have no competing interests.

### Reviewer's report 3

#### Zhenguo Lin, Department of Ecology and Evolution, The University of Chicago (nominated by John Logsdon)

Different organisms may respond differently to the specific stress. It is of great interest to study how such difference was evolved by examining the evolution of the related stress response pathways. The yeast *S. cerevisiae *cells respond to increased intracellular osmolarity by activating of the high osmolarity glycerol (HOG) MAPK pathway. This manuscript indentify the orthologous genes of HOG pathway from 10 closely related yeast species, and examined the evolutionary rates of genes at each step of this pathway. The authors also investigated the other factors that may affect the evolutionary rates of these genes. The authors found that the HOG pathway is conserved in hemiascomycete yeasts, but the evolutionary rates vary greatly in different lineages. One interesting observation is that the evolutionary rates of genes tend to be negatively correlated with their positions in the pathway, suggesting a stronger selection constrains at the downstream genes. Overall, this is a well-executed analysis and well organized manuscript, although more work need to be done to improve the quality of writing.

Major comments

1. The authors claimed that the *MSB2 *genes have been under positive selection. Although the ω = 0.2496 value is probably the largest among all genes in the pathway, it is still far below 1. However, if *MSB2 *was under positive selection does not affect the conclusion of this paper.

Author's response: *Although the d_N_/d_S _value of the whole sequence of MSB2 is below 1, but some sites (codons) of this gene may evolve fast and under positive selection. We used the site model M7 and M8 comparison to detect positively selected genes, which allow d_N_/d_S _to vary among codons*.

2. In the introduction, the authors said *C. albicans *and *A. gossypii *have different sensitivity responding to environmental osmolarity stress. Are the evolutionary rates of HOG genes different between the two species? It would make this study more valuable if the results are correlated with different osmolarity stress responses among different species.

Author's response: *Thanks for the reviewer's useful comments. We did observe the difference of evolutionary rates of these two species (Wilcoxon rank sum test, P = 0.00213). However, the omostic stress resistance have been compared in only a few species, and the resistance capability cannot be quantified, so we cannot analysis the correlation relationship between evolutionary rate and stress resistance quantitatively*.

3. The Figure 5 shows a negative correlation between the *d*_N_/*d*_S _value and the position during the HOG signal transduction process. As said by authors, the evolutionary rates are quite different among different lineages. I am not sure if it is a good idea to mix data from all lineages to study the correlation. Does the correlation still exist in each individual species or how many species have such pattern?

Author's response: *Following the reviewer's suggestion, we have considered evolutionary rate of each lineage of the surveyed yeast taxa, and found the pattern of d_N_/d_S _variation correlated significantly with pathway position still holds in S. parodoxus, S. miketae and A. gossypii*.

Minor comments

" are characterized extensively" → "have been characterized extensively"

Author's response:*Following the reviewer's suggestion, we haved changed " are characterized extensively" to "have been characterized extensively"*.

"10 yeast genomes" → "10 yeast species"

Author's response: *Following the reviewer's suggestion, we haved changed " 10 yeast genomes " to "10 yeast species "*.

"Among them" is not clear to the context. "them" could be misleading as the three functional modules.

Author's response: *Following the reviewer's suggestion, we have removed these two words*.

fungi is more "evolutionary conserved" is that true? any reference?

Author's response: *Following the reviewer's suggestion, we have added two references*."proofed" proof is not a verb

Author's response: *Following the reviewer's suggestion, we have changed " proofed " to " proved "*.

"The aligned amino acid sequences were reversely translated into nucleotide sequences by in-house Perl script for further analysis". Did you mean convert protein alignment into DNA alignment using their original DNA sequence? It is impossible to "Translate" amino acid sequences to translated nucleotide.

Author's response: *Following the reviewer's suggestion, we have changed " translated " to " converted "*.

" we generated 19 HOG-MAPK pathway genes" it is not appropriate to use "generated" here.

Author's response: *We agree with the reviewer and have changed "generate" to "identified"*.

" their surrounding genes" it is misleading. it could be neighboring genes on the chromosome.

Author's response: *Following the reviewer's suggestion, we have add "in HOG pathway" in this sentence*.

" Several earlier studies", only one reference is listed.

Author's response: *Following the reviewer's suggestion, we have added two references*.

"fungi" → fungal

Author's response: *Following the reviewer's suggestion, we have changed "fungi " to " fungal "*.

I declare that I have no competing interests.

## References

[B1] Bahn YS Xue C Idnurm A Rutherford JC Heitman J Cardenas ME Sensing the environment: lessons from fungi Nat Rev Microbiol 2007 5 57 69 10.1038/nrmicro1578 17170747

[B2] Gasch AP Comparative genomics of the environmental stress response in ascomycete fungi Yeast 2007 24 961 976 10.1002/yea.1512 17605132

[B3] Singh AH Wolf DM Wang P Arkin AP Modularity of stress response evolution Proc Natl Acad Sci USA 2008 105 7500 7505 10.1073/pnas.0709764105 18495925 PMC2396705

[B4] Jin Y Weining S Nevo E A MAPK gene from Dead Sea fungus confers stress tolerance to lithium salt and freezing-thawing: Prospects for saline agriculture Proc Natl Acad Sci USA 2005 102 18992 18997 10.1073/pnas.0509653102 16365289 PMC1323214

[B5] Westfall PJ Ballon DR Thorner J When the stress of your environment makes you go HOG wild Science 2004 306 1511 1512 10.1126/science.1104879 15567851

[B6] Chen RE Thorner J Function and regulation in MAPK signaling pathways: lessons learned from the yeast *Saccharomyces cerevisiae* Biochim Biophys Acta 2007 1773 1311 1340 10.1016/j.bbamcr.2007.05.003 17604854 PMC2031910

[B7] Krantz M Becit E Hohmann S Comparative genomics of the HOG-signalling system in fungi Curr Genet 2006 49 137 151 10.1007/s00294-005-0038-x 16468042

[B8] O'Rourke S Herskowitz I Unique and redundant roles for HOG MAPK pathway components as revealed by whole-genome expression analysis Molecular Biology of the Cell 2004 15 532 542 10.1091/mbc.E03-07-0521 14595107 PMC329229

[B9] Hersen P McClean MN Mahadevan L Ramanathan S Signal processing by the HOG MAP kinase pathway Proc Natl Acad Sci USA 2008 105 7165 7170 10.1073/pnas.0710770105 18480263 PMC2386076

[B10] Nikolaou E Agrafioti I Stumpf M Quinn J Stansfield I Brown AJ Phylogenetic diversity of stress signalling pathways in fungi BMC Evol Biol 2009 9 44 10.1186/1471-2148-9-44 19232129 PMC2666651

[B11] Wolf MY Wolf YI Koonin EV Comparable contributions of structural-functional constraints and expression level to the rate of protein sequence evolution Biol Direct 2008 3 40 10.1186/1745-6150-3-40 18840284 PMC2572155

[B12] Ingvarsson PK Gene expression and protein length influence codon usage and rates of sequence evolution in *Populus tremula* Mol Biol Evol 2007 24 836 844 10.1093/molbev/msl212 17204548

[B13] Graur D Li W Fundamentals of molecular evolution 2000 Sunderland, MA: Sinauer Associates

[B14] Alvarez-Ponce D Aguade M Rozas J Network-level molecular evolutionary analysis of the insulin/TOR signal transduction pathway across 12 *Drosophila *genomes Genome Res 2009 19 234 242 10.1101/gr.084038.108 19141596 PMC2652205

[B15] Yang YH Zhang FM Ge S Evolutionary rate patterns of the Gibberellin pathway genes BMC Evol Biol 2009 9 206 10.1186/1471-2148-9-206 19689796 PMC2794029

[B16] Beskow A Wright AP Comparative analysis of regulatory transcription factors in *Schizosaccharomyces pombe *and budding yeasts Yeast 2006 23 929 935 10.1002/yea.1413 17072884

[B17] Kellis M Birren BW Lander ES Proof and evolutionary analysis of ancient genome duplication in the yeast *Saccharomyces cerevisiae* Nature 2004 428 617 624 10.1038/nature02424 15004568

[B18] Li YD Liang H Gu Z Lin Z Guan W Zhou L Li YQ Li WH Detecting positive selection in the budding yeast genome J Evol Biol 2009 22 2430 2437 10.1111/j.1420-9101.2009.01851.x 19878412

[B19] Jovelin R Dunham JP Sung FS Phillips PC High nucleotide divergence in developmental regulatory genes contrasts with the structural elements of olfactory pathways in *caenorhabditis* Genetics 2009 181 1387 1397 10.1534/genetics.107.082651 19001295 PMC2666507

[B20] Jovelin R Phillips PC Evolutionary rates and centrality in the yeast gene regulatory network Genome Biol 2009 10 R35 10.1186/gb-2009-10-4-r35 19358738 PMC2688926

[B21] Sackton TB Lazzaro BP Schlenke TA Evans JD Hultmark D Clark AG Dynamic evolution of the innate immune system in *Drosophila* Nat Genet 2007 39 1461 1468 10.1038/ng.2007.60 17987029

[B22] Muzzey D Gomez-Uribe CA Mettetal JT van Oudenaarden A A systems-level analysis of perfect adaptation in yeast osmoregulation Cell 2009 138 160 171 10.1016/j.cell.2009.04.047 19596242 PMC3109981

[B23] Rausher M Miller R Tiffin P Patterns of evolutionary rate variation among genes of the anthocyanin biosynthetic pathway SMBE 1999 16 266 274 10.1093/oxfordjournals.molbev.a026108 10028292

[B24] Ramsay H Rieseberg LH Ritland K The correlation of evolutionary rate with pathway position in plant terpenoid biosynthesis Mol Biol Evol 2009 26 1045 1053 10.1093/molbev/msp021 19188263

[B25] O'Rourke S Herskowitz I O'Shea E Yeast go the whole HOG for the hyperosmotic response Trends in Genetics 2002 18 405 412 10.1016/S0168-9525(02)02723-3 12142009

[B26] Pokholok DK Zeitlinger J Hannett NM Reynolds DB Young RA Activated signal transduction kinases frequently occupy target genes Science 2006 313 533 536 10.1126/science.1127677 16873666

[B27] Cherry JM Adler C Ball C Chervitz SA Dwight SS Hester ET Jia Y Juvik G Roe T Schroeder M Weng S Botstein D SGD: *Saccharomyces *Genome Database Nucleic Acids Res 1998 26 73 79 10.1093/nar/26.1.73 9399804 PMC147204

[B28] Arnaud MB Costanzo MC Skrzypek MS Binkley G Lane C Miyasato SR Sherlock G The *Candida *Genome Database (CGD), acommunity resource for *Candida albicans *gene and protein information Nucleic Acids Res 2005 33 D358 363 10.1093/nar/gki003 15608216 PMC539957

[B29] Byrne KP Wolfe KH The Yeast Gene Order Browser: combining curated homology and syntenic context reveals gene fate in polyploid species Genome Res 2005 15 1456 1461 10.1101/gr.3672305 16169922 PMC1240090

[B30] Kellis M Patterson N Endrizzi M Birren B Lander ES Sequencing and comparison of yeast species to identify genes and regulatory elements Nature 2003 423 241 254 10.1038/nature01644 12748633

[B31] Li YD Xie ZY Du YL Zhou Z Mao XM Lv LX Li YQ The rapid evolution of signal peptides is mainly caused by relaxed selection on non-synonymous and synonymous sites Gene 2009 436 8 11 10.1016/j.gene.2009.01.015 19393172

[B32] Larkin MA Blackshields G Brown NP Chenna R McGettigan PA McWilliam H Valentin F Wallace IM Wilm A Lopez R Thompson JD Gibson TJ Higgins DG Clustal W and Clustal X version 2.0 Bioinformatics 2007 23 2947 2948 10.1093/bioinformatics/btm404 17846036

[B33] Hall T BioEdit: a user-friendly biological sequence alignment editor and analysis program for Windows 95/98/NT Nucleic Acids Symposium Series 1999 41 95 98

[B34] Rokas A Williams BL King N Carroll SB Genome-scale approaches to resolving incongruence in molecular phylogenies Nature 2003 425 798 804 10.1038/nature02053 14574403

[B35] Ronquist F Huelsenbeck JP MrBayes 3: Bayesian phylogenetic inference under mixed models Bioinformatics 2003 19 1572 1574 10.1093/bioinformatics/btg180 12912839

[B36] Yang Z PAML 4: phylogenetic analysis by maximum likelihood Mol Biol Evol 2007 24 1586 1591 10.1093/molbev/msm088 17483113

[B37] Wong WS Yang Z Goldman N Nielsen R Accuracy and power of statistical methods for detecting adaptive evolution in protein coding sequences and for identifying positively selected sites Genetics 2004 168 1041 1051 10.1534/genetics.104.031153 15514074 PMC1448811

[B38] Goldman N Yang Z A codon-based model of nucleotide substitution for protein-coding DNA sequences Mol Biol Evol 1994 11 725 736 7968486 10.1093/oxfordjournals.molbev.a040153

[B39] Connallon T Knowles LL Recombination rate and protein evolution in yeast BMC Evol Biol 2007 7 235 10.1186/1471-2148-7-235 18042299 PMC2211315

